# Development of the caudal-fin skeleton reveals multiple convergent fusions within Atherinomorpha

**DOI:** 10.1186/s12983-021-00408-x

**Published:** 2021-04-26

**Authors:** Philipp Thieme, Peter Warth, Timo Moritz

**Affiliations:** 1 Deutsches Meeresmuseum, Katharinenberg 14–20, 18439 Stralsund, Germany; 2 Institut für Zoologie und Evolutionsforschung, Friedrich-Schiller-Universität Jena, Erbertstraße 1, 07743 Jena, Germany; 3 Staatliches Museum für Naturkunde Stuttgart, Rosenstein 1, 70191 Stuttgart, Germany

**Keywords:** Ontogeny, Atheriniformes, Beloniformes, Cyprinodontiformes, Compound centrum, Hypural plate, Morphology

## Abstract

**Background:**

The caudal fin of teleosts is a highly diverse morphological structure and a valuable source of information for comparative analyses. Within the Atherinomorpha a high variation of conditions of the caudal-fin skeleton can be found. These range from complex but basal configurations to simple yet derived configurations. When comparing atherinomorph taxa, it is often difficult to decide on the homology of skeletal elements if only considering adult specimens. However, observing the development of caudal-fin skeletons allows one to evaluate complex structures, reveal homologies and developmental patterns, and even reconstruct the grundplan of the examined taxa.

**Results:**

We studied the development of the caudal-fin skeleton in different atheriniform, beloniform and cyprinodontiform species using cleared and stained specimens. Subsequently we compared the development to find similarities and differences in terms of 1) which structures are formed and 2) which structures fuse during ontogeny. For many structures, i.e., the parhypural, the epural(s), the haemal and neural spines of the preural centra and the uroneural, there were either no or only minor differences visible between the three taxa. However, the development of the hypurals revealed a high variation of fusions within different taxa that partly occurred independently in atheriniforms, beloniforms and cyprinodontiforms. Moreover, comparing the development of the ural centra exposed two ways of formation of the compound centrum: 1) in atheriniforms and the beloniforms *Oryzias* and *Hyporhamphus limbatus* two ural centra develop and fuse during ontogeny while 2) in cyprinodontiforms and Exocoetidae (Beloniformes) only a single ural centrum is formed during ontogeny.

**Conclusions:**

We were able to reconstruct the grundplan of the developmental pattern of the caudal-fin skeleton of the Atheriniformes, Beloniformes and Cyprinodontiformes as well as their last common ancestors. We found two developmental modes of the compound centrum within the Atherinomorpha, i.e., the fusion of two developing ural centra in atheriniforms and beloniforms and the development of only one ural centrum in cyprinodontiforms. Further differences and similarities for the examined taxa are discussed, resulting in the hypothesis that the caudal-fin development of a last common ancestor to all atherinomorphs is very much similar to that of extant atheriniforms.

## Background

Compared to other fish-like vertebrates, teleosts have a highly specialized caudal fin and, starting from a common bauplan, the caudal-fin skeleton evolved a high morphological diversity within Teleostei [1–3]. Sometimes the morphological diversity is very high within certain teleostean taxa, e.g. Osteoglossomorpha [4] or Paracanthopterygii [5]. Morphological studies of phylogenetic relationships of teleosts therefore often use the caudal-fin skeleton as a rich source of information [6–9]. Also within the Atherinomorpha, comprising the Atheriniformes, Beloniformes and Cyprinodontiformes [10], an immense variety is present, ranging from a presumably basal condition, with distinct hypurals, e.g. in *Odontesthes bonariensis* ([1]: Fig. 168), to taxa in which most of the caudal-fin skeleton is fused into one large compound structure, e.g. in *Hypsolebias trilineatus* ([11]: Fig. 3). The evolution of the caudal-fin skeleton within atherinomorphs however is not well understood and requires further study, especially since in the light of current phylogenetic hypotheses, fusions and losses of different elements appear to have happened multiple times independently within the group.

The monophyly of the Atherinomorpha is widely accepted and was first suggested almost 60 years ago, based on various character similarities, e.g. absence of pharyngobranchial 1 and attachment of Baudelot’s ligament to the basicranium [10], which are both shared by other taxa. In subsequent morphological phylogenetic analyses, the monophyly of atherinomorphs was confirmed multiple times [12–21] and synapomorphies such as “rostral cartilage decoupled from premaxilla” or “the absence of the third, fourth and fifth infraorbital” have been proposed ([17], pp., 20-21). Many, especially recent, molecular analyses also support the close relationship of atheriniforms, beloniforms and cyprinodontiforms [22–29]. In the past decades, only few studies questioned the monophyly of Atherinomorpha by including representatives of other taxa, i.e. mugilids, cichlids, blenniids and gobiesocids, though mostly with little support [30–33]. In the latest molecular studies, all these taxa as well as Atherinomorpha and many other taxa are positioned in the recently proposed Ovalentaria [26–29]. The taxon Ovalentaria is well supported by large amounts of molecular data, but the relationships within the Ovalentaria presently remain obscure. This complicates outgroup comparisons for atherinomorph characters. However, the proposed assemblage of taxa offers new impulses for comparative analyses and will be used as working hypothesis in the present study.

Within the Atherinomorpha, the Atheriniformes are considered to be the earliest branching taxon while the Beloniformes and Cyprinodontiformes form a sister-clade and are regarded more derived [17, 19, 22, 24, 25, 27]. This view is challenged by recent studies based on large molecular datasets: Betancur-R R, Wiley EO, Arratia G*,* et al. [28] and Hughes LC, Orti G, Huang Y*,* et al. [29] proposed that beloniforms are the earliest branching taxon within atherinomorphs and atheriniforms and cyprinodontiforms are more derived sister taxa. However, morphological characters clearly support the basal position of atheriniforms which in many character complexes show the more basal condition, while beloniforms and cyprinodontiforms share reduced or fused conditions, e.g., further reduction of infraorbitals or the absence of the first pharyngobranchial, that are regarded more derived [10, 13, 14, 16, 17, 19, 20]. The caudal skeleton of atherinomorphs however is not understood well enough to draw an evolutionary scenario in the light of this phylogenetic framework. We therefore analysed the caudal-fin skeleton of several ovalentarian taxa for comparison and especially the development of the caudal-fin skeleton in representatives of Beloniformes, Cyprinodontiformes and Atheriniformes.

Developmental morphology is a powerful scientific approach to infer homology of elements and uncover apomorphic characters (e.g. [34]). In the present study, we investigated the development of the caudal-fin skeleton within all subgroups of atherinomorphs allowing detailed evaluation of the complex anatomy of the caudal-fin skeleton found in several adult atherinomorph taxa. The results allow us to trace the evolution of caudal-fin development within this taxon, revealing homologous and convergent developmental patterns, and allowing us to reconstruct the grundplan of the Atherinomorpha and its comprising taxa Atheriniformes, Beloniformes and Cyprinodontiformes.

## Material and methods

### Larval rearing and sampling

Fish larvae of the species *Aplocheilus lineatus*, *Epiplatys annulatus*, *Glossolepis incisus*, *Oryzias woworae*, *Poropanchax normani* and *Pseudomugil furcatus* were reared at the facilities of the Deutsches Meeresmuseum in Stralsund, Germany. Fertilized eggs were collected constantly once per week from spawning mops, which were placed in each species tank respectively, and raised at room temperature (23–24 °C), consistent water conditions of 400–500 μS, and pH 7.2–7.5. For *A. lineatus*, *E. annulatus*, *O. woworae* and *Po. normani* first samples were taken before hatching occurred and there the eggshell was removed before further steps proceeded. All sampled specimens were euthanised using a benzocain-solution (Ethyl p-Amino Benzoate, E-1501, Sigma Aldrich, MO, USA). Afterwards they were fixed in 4% formaldehyde.

Specimens of *Hyporhamphus* cf. *limbatus* were sampled with a 500 μm mesh plankton net in mangrove creek channels in the Persian Gulf. The net was towed from a small boat at low speed for 5 to 10 min per tow. Larvae were immediately fixed in formaldehyde and later transferred to 70% ethanol for long term storage at the Phyletisches Museum, Jena.

### Clearing and staining

Specimens examined in this study were either reared (as stated above) at the Deutsches Meeresmuseum, taken from the ichthyological collection of the Deutsches Meeresmuseum, Stralsund (Table 1) or taken from the collection of the Phyletisches Museum, Jena (Table 1). Clearing and staining of the specimens principally followed the protocols of Dingerkus G and Uhler LD [35], Schnell NK, Konstantinidis P and Johnson GD [36] and Taylor WR and Van Dyke GC [37]. Reared embryos and larvae were transferred to 100% ethanol after fixation using an ascending ethanol series (30, 50, 70%). Collection material, which was stored in 70% ethanol, was directly transferred to 100% ethanol for clearing and staining. Afterwards specimens were stained for cartilage using an Alcian blue solution (2 parts glacial acetic acid and 8 parts 100% ethanol with 0.04 g/100 ml Alcian blue powder). Specimens were placed in this solution until the distal radials of the anal pterygiophores were stained distinctly blue, which took up to 3 h for embryos and larvae and up to 16 h for adults. Before clearing, the specimens were put back into 100% ethanol and then transferred to a borate-solution (65 to 35% saturated borate solution/distilled water) via a descending ethanol series (70, 50, 30%). A trypsin solution was used for clearing (0,0375 g trypsin powder [1000–2000 BAEE units/mg, Sigma Aldrich, MO, USA] per 100 ml diluted borax solution) of the specimens. Depending on size, it took up to 8 h for embryos and larvae to clear, while adults took up to 5 days. For bleaching, the specimens were placed in a 0.5% KOH solution to which 0.05 ml 30% H_2_O_2_ was added per 100 ml. After removal of all pigments, the specimens were transferred into an Alizarin red solution (0.01 g Alizarin red powder per 100 ml 0.5% KOH) for bone staining. Lastly, the specimens were transferred into 1:2, 1:1 and 2:1 solutions of 100% glycerol to 0.5% KOH before being placed in 86.5% glycerol for documentation and storage.

**Table 1:** List of specimens from the Deutsches Meeresmuseum (DMM), Phyletisches Museum (PMJ) and Zoologisches Forschungsmuseum Alexander König (ZFMK) examined during this study. Length as standard length (SL) and as notochord length (NL, indicated by asterisk)

Taxon	Species	Collection ID	number of specimens	length
Atheriniformes	*Atherina hepsetus*	DMM IE/11378	1	31.59
		DMM IE/11405	1	36.49
		DMM IE/16510	2	59.48–70.32
	*Atherina presbyter*	DMM IE/11387	16	8.12–12.54
		DMM IE/14967	1	21.46
		DMM IE/14969	71	9.80–16.38
	*Bedotia geayi*	DMM IE/11396	12	5.38–15.83
		DMM IE/11397	5	3.22*-6.55
		DMM IE/11411	4	4.64*-8.39
		DMM IE/15880	2	62.40–78.11
		DMM IE/16309	2	56.28–63.13
		DMM IE/16583	7	4.86*-10.49
		DMM IE/16590	7	6.61–19.44
	*Glossolepis incisus*	DMM IE/12202	1	45.73
		DMM IE/15953	1	57.72
		DMM IE/16585	33	2.49*-10.48
	*Iriatherina werneri*	DMM IE/16589	19	4.73*-11.44
	*Leuresthes tenuis*	DMM IE/16591	16	2.28*-4.44
	*Marosatherina ladigesi*	DMM IE/11011	6	19.86–33.98
		DMM IE/11388	13	4.34*-11.79
		DMM IE/11389	8	4.25*-10.53
		DMM IE/11390	8	4.68*-9.81
		DMM IE/11402	1	48.13
		DMM IE/11413	6	3.27*-8.90
	*Melanotaenia lacustris*	DMM IE/11375	8	3.36*-13.48
		DMM IE/11376	8	5.56–13.69
		DMM IE/11379	2	12.29–13.51
		DMM IE/11414	7	6.37–14.55
		DMM IE/12226	1	56.52
		DMM IE/16533	1	48.48
		DMM IE/16593	8	7.03–10.59
	*Membras martinica*	DMM IE/11398	1	49.01
	*Menidia conchorum*	DMM IE/11399	1	65.51
	*Odonthestes bonariensis*	DMM IE/14958	1	50.66
	*Pseudomugil furcatus*	DMM IE/16310	1	41.17
		DMM IE/16311	1	41.14
		DMM IE/16314	21	3.18*-14.71
		DMM IE/16315	11	4.30–5.99
		DMM IE/16582	12	3.83*-11.48
	*Pseudomugil paskai*	DMM IE/11380	1	27.90
	*Pseudomugil signifer*	DMM IE/11408	1	24.03
Beloniformes	*Belone belone*	DMM IE/16512	23	25.27–30.40
		DMM IE/16519	1	84.72
	*Dermogenys pusilla*	DMM IE/16534	1	37.45
	*Dermogenys* cf. *siamensis*	DMM IE/16502	5	23.67–27.15
	*Hyporhamphus* cf. *limbatus*	PMJ PISC-1857	10	7.71–10.81
		PMJ PISC-1857	5	5.65–15.07
		PMJ PISC-1857	1	3.65*
		PMJ PISC-1857	2	11.91–20.07
	*Nomorhamphus kolonidalensis*	ZFMK 49237–53	1	29.08
	*Oryzias sinensis*	DMM IE/16499	10	13.86–21.16
	*Oryzias woworae*	DMM IE/16527	1	20.43
		DMM IE/16530	3	6.78–10.87
		DMM IE/16587	92	2.23*-10.74
	*Xenentodon cancila*	DMM IE/16509	1	95.66
Cyprinodontiformes	*Ameca splendens*	DMM IE/16535	1	37.91
	*Anableps microlepis*	DMM IE/14934	4	53.49–68.39
	*Aphysoemion bitaeniatum*	DMM IE/16522	1	20.93
	*Aphyosemion striatum*	DMM IE/16581	14	1.93*-3.50
	*Aplocheilus lineatus*	DMM IE/16584	15	3.45*-7.23
		DMM IE/16599	2	34.42–42.08
	*Epiplatys annulatus*	DMM IE/16588	12	1.80*-3.73
	*Epiplatys sexfasciatus*	DMM IE/16470	1	29.80
	*Epiplatys spilargyreius*	DMM IE/14947	3	17.78–21.20
	*Nothobranchius eggersi*	DMM IE/16597	5	3.67–5.62
	*Pachypanchax omalonotus*	DMM IE/11392	17	4.45–14.31
		DMM IE/11403	1	47.20
		DMM IE/11410	4	5.58–6.59
		DMM IE/16596	10	4.43–6.16
	*Poropanchax normani*	DMM IE/16525	2	15.08–16.12
		DMM IE/16586	30	1.98*-4.40
	*Poecilia sphenops*	DMM IE/12198	3	36.52–57.69
		DMM IE/16594	4	6.51–7.43
		DMM IE/16595	9	5.68–7.12
Cichlidae	*Amatitlania nigrofasciata*	DMM IE/16598	18	4.97–8.63
	*Geophagus* sp.	IE/15931	6	4.96–5.60
		IE/15932	6	4.59–5.47
	*Hemichromis bimaculatus*	IE/16592	10	4.97–12.83
Pomacentridae	*Amphiprion ocellaris*	IE/11382	14	2.94*-9.65
		IE/11383	7	3.56*-4.38

The caudal fin-development was analysed in multiple atheriniform, beloniform and cyprinodontiform species. Developmental series of seven atheriniform species (i.e., *Atherina presbyter*, *Bedotia geayi*, *Glossolepis incisus*, *Iriatherina werneri*, *Leuresthes tenuis*, *Melanotaenia lacustris*, and *Pseudomugil furcatus*), two beloniform species (*Hyporhamphus* cf. *limbatus*, *Oryzias woworae*), and six cyprinodontiform species (*Aphyosemion striatum*, *Aplocheilus lineatus*, *Epiplatys annulatus*, *Pachypanchax omalonotus*, *Poropanchax normani*, *Poecilia sphenops*) were examined using cleared and stained specimens (Table 1). Further, larval, juvenile, and adult specimens of eighteen additional species from all three taxa were evaluated (Table 1). For comparison, developmental series of three cichlid species and one pomacentrid species were studied (Table 1).

### Imaging and documentation

For documentation of the caudal-fin development, embryos and larvae were photographed using a Leica M205 FCA with an attached Leica DMC6200 camera operated with the software Leica Application Suite (version: 3.6.0.20104). Additionally, specimens of *Glossolepis incisus*, *Oryzias woworae* and *Poropanchax normani* were imaged using fluorescent light making use of the autofluorescent properties of Alizarin red. Adult specimens were photographed using a Canon EOS 80D with a Canon MP-E 65 mm objective. Images were processed, without altering any morphological structures, and drawings were produced using Adobe Photoshop (version: 22.0.0). Figure plates were assembled in Adobe Illustrator (version: 25.0).

### Terminology

The terminology used in this study in general follows the definitions given in Fujita K [38] and Schultze H-P and Arratia G [8]. Differing from the latter, we herein define the compound centrum as the most posterior vertebral centrum to which the lower and upper hypurals and the parhypural are connected (either fused to the vertebral centrum or articulating with it). The term does not infer any homology of the structure across taxa. Numbering of elements, e.g., the epurals, does not necessarily reflect the assumption of homology across taxa or an attribution to a certain body segment. List of abbreviations: CC, compound centrum; EO, extra caudal ossicle; EU, epural; HA, haemal arch; HP, hypural plate; HS, haemal spine; HYP, hypural; IHC, inter-haemal spine cartilage; INC, inter-neural spine cartilage; LHP, lower hypural plate; NA, neural arch; NO, notochord; NS, neural spine; OPC, opisthural cartilage; PH, parhypural; PU, preural centrum; UC, ural centrum; UHP, upper hypural plate; UN, uroneural.

## Results

### Atheriniformes

#### Melanotaeniidae: *Glossolepis incisus* (Figs. 1a, and 2)

**Fig. 1:**
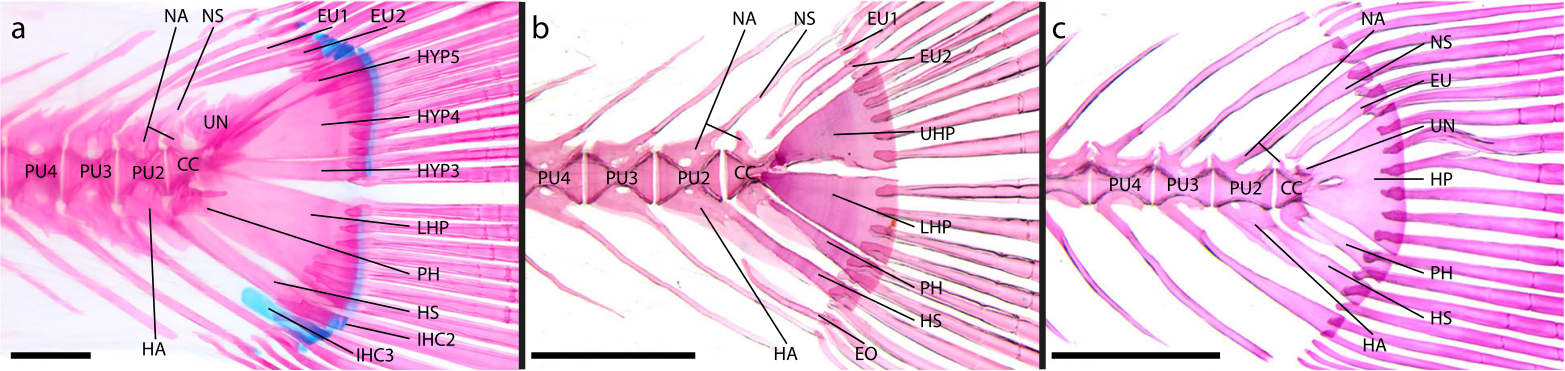
Adult caudal-fin skeleton of **a** *Glossolepis incisus*, DMM IE/12202 SL = 45.7 mm; **b** *Oryzias woworae*, DMM IE/16527 SL = 20.43 mm; **c** *Poropanchax normani*, DMM IE/16525 SL = 16.12 mm. Abbreviations: CC, compound centrum; EO, extra caudal ossicle; EU, epural; HA, haemal arch; HP, hypural plate; HS, haemal spine; HYP, hypural; IHC, inter-haemal spine cartilage; LHP, lower hypural plate; NA, neural arch; NS, neural spine; PH, parhypural; PU, preural centrum; UN, uroneural. Scale bar: 1 mm

The caudal-fin skeleton of *G. incisus* (Fig. 1a) comprises the compound centrum (CC) and the preural centra 2, 3, 4, and 5 (PU2–5) as well as the associated dorsal (except PU5) and ventral elements. Fused to each of the PU are a neural arch dorsally and a haemal arch ventrally with an elongated neural (NS) and a haemal spine (HS) respectively. The NS of PU2 is exceptional, as it is only about one third of the length of the other neural spines in the caudal region. The HS of PU2 is slightly broadened in lateral view. The shape of the CC is characterized by a half-hourglass shaped anterior portion and an upward-pointing posterior cone-like portion The PH and the LHP are almost completely fused, with only a small gap remaining proximally, where they approach the CC. While the proximal part of the PH articulates with the anterior portion of the CC, the LHP is firmly fused to the CC posteriorly. Posterodorsally, hypural (HYP) 3, HYP4 and HYP5 articulate to the CC. HYP4 and HYP5 are fused along a well visible margin. Membranous extensions of the CC overlap the anterior HYP3 and HYP4 laterally. A reduced neural arch is fused dorsally to the anterior portion of the CC. One pair of uroneurals (UN) is present dorsal to the posterior portion of the CC and overlaps with HYP5 laterally. Between the NS of PU3 and HYP5, two epurals (EU) are present. Posterior to the distal tip of the HS of PU2 and between the distal tips of the HS of PU2 and PU3 the inter-haemal spine cartilage (IHC) 2 and 3 are present respectively.

**Fig. 2:**
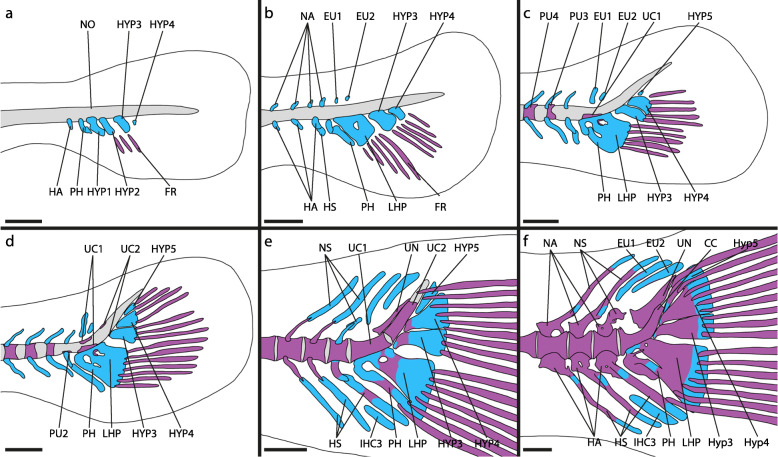
Development of the caudal-fin skeleton of the atheriniform *Glossolepis incisus* (DMM IE/16585). **a** NL = 4.86 mm; **b** SL = 6.13 mm; **c** SL = 6.56 mm; **d** SL = 6.48 mm; **e** SL = 9.50 mm; **f** SL = 15.01 mm. Abbreviations: CC, compound centrum; EU, epural; FR, fin ray; HA, haemal arch; HS, haemal spine; HYP, hypural; IHC, inter-haemal spine cartilage; LHP, lower hypural plate; NA, neural arch; NO, notochord; NS, neural spine; PH, parhypural; PU, preural centrum; UC, ural centrum; UN, uroneural. Scale bar: 200 μm

**Fig. 3:**
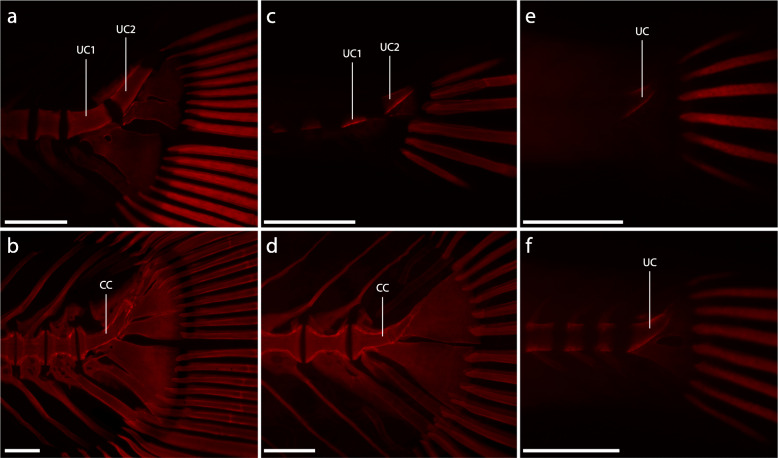
Development of the ural centra of *Glossolepis incisus* (**a, b**), *Oryzias woworae* (**c, d**) and *Poropanchax normani* (**e, f**) visualized with Alizarin-red autofluorescence. **a** DMM IE/16585, SL = 7,76 mm; **b** DMM IE/16585, SL = 15.01 mm; **c** DMM IE/16587, SL = 3.78 mm; **d** DMM IE/16587, SL = 7.28 mm; **e** DMM IE/16586, SL = 3.32 mm; **f** DMM IE/16586, SL = 3.43 mm. Abbreviations: CC, compound centrum; UC, ural centrum. Scale bar: 200 μm

The development of the caudal-fin skeleton of *Glossolepis incisus* starts with the appearance of cartilaginous precursors to the PH and HYP1, HYP2, HYP3 and HYP4 (Fig. 2a). At this stage already three principal-fin rays are distinguishable. While the first vertebral centra start to ossify from anterior to posterior, the haemal arches and neural arches develop beforehand in the same direction. However, the neural arches develop slightly after the haemal arches. The haemal arch of PU2 emerges after the PH and HYP1–4 are developed (Fig. 2b). Shortly after their appearance, the cartilaginous HYP1 and HYP2 fuse distally and later also proximally, forming the LHP (Fig. 2b). Proximal within the LHP a foramen persists. Five ventral and five dorsal principal-fin rays can be distinguished. The cartilaginous precursors to EU1 and EU2 as well as the neural arch of PU2 form next (Fig. 2b). Flexion of the notochord starts only after the onset of the development of these structures. Between the distal tips of the haemal arch of PU2 the associated haemal spine appears as a small autogenous cartilage. During ontogeny it enlarges gradually in ventral direction. The cartilaginous precursor to HYP5 appears dorsal to HYP4 (Fig. 2c). A cartilaginous connection between the proximal tip of the PH and the LHP is established. Also, the PH fuses distally to the LHP (Fig. 2c). Antero-dorsally to the LHP an ossification centre develops around the ventral surface of the notochord (Fig. 2c). This ossification centre represents ural centrum (UC) 1 and subsequently grows dorsally. Opposite to the first ossification centre on the notochord another one emerges and both grow towards each other to form a full centrum (Fig. 2d). There are now seven ventral and seven dorsal principal-fin rays present. The vertebral centrum of PU2 forms next. First, an ossification centre emerges ventrally and later also dorsally. Anterior to HYP3 and HYP4 a ventral and a dorsal ossification centre develop around the notochord representing UC2. These ossifications also grow towards each other to form a full vertebral centrum (Fig. 3a). Ossification of the hypurals begins at the antero-dorsal portion of HYP1 (Fig. 2d). While HYP1 then gradually ossifies from anterior to posterior, ossification sites appear in all other hypurals and the PH and they too ossify from anterior to posterior (Fig. 2e). Anterior to UC2 the paired uroneural develops and then elongates in ventral and dorsal direction. The autogenous haemal spine of PU2 also ossifies in this stage. Anterior to its distal tip a cartilage emerges, the IHC3. The epurals start to ossify from the middle to the tips. The margins of the two ural centra get close together and fusion of these two centra starts (Fig. 2f). UC2 then gets shorter and a CC is formed (Fig. 2f, 3b). HYP4 and HYP5 first fuse distally, then proximal so that a foramen is formed, which later is reduced due to complete fusion of the two hypurals. The boundaries of each hypural nevertheless remain visible even in adults (Fig. 1a). The LHP starts to fuse to the CC. Proximally on the PH the parhypurapophysis develops. The proximal cartilaginous part of the PH, connecting it to the LHP gets reduced and the PH grows proximally around the CC to which it then articulates. After all elements of the caudal-fin skeleton have formed and most of them are ossified, the CC shrinks relative to the other elements, as the dorsal/posterior portion is reduced to a short upwards-directed horn. The uroneural grows dorsally and overlies the HYP4 and HYP5 laterally. From the CC a triangular outgrowth is formed, which covers the proximal margin of HYP3 laterally (Fig. 1a).

#### Other Atheriniformes

**Fig. 4:**
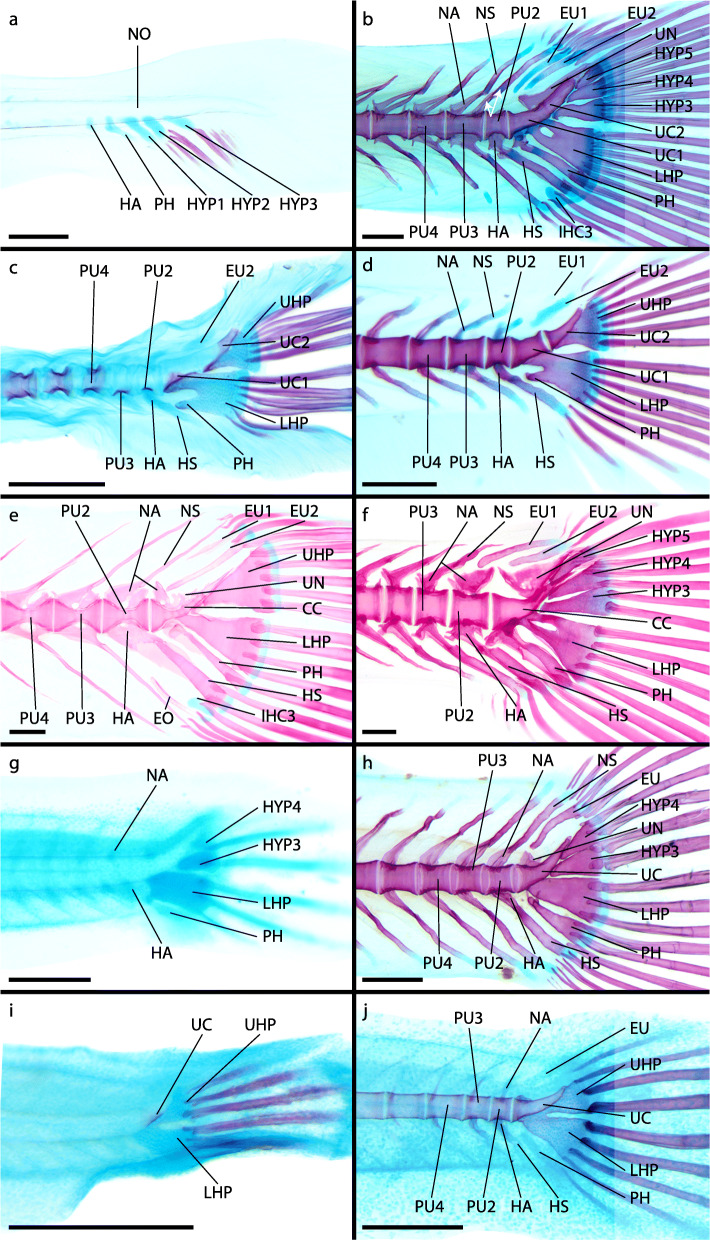
Developmental stages of additional atheriniform (**a-d**), beloniform (**e, f**) and cyprinodontiform (**g-j**) species. **a** *Bedotia geayi*, DMM IE/16583, NL = 4.86 mm; **b** *B. geayi*, DMM IE/16583, SL = 10.49 mm; **c** *Pseudomugil furcatus*, DMM IE/16582, SL = 3.38 mm; **d** *P. furcatus*, DMM IE/16582, SL = 5.89 mm; **e** *Oryzias sinensis*, DMM IE/16499 SL = 13.86 mm; **f** *Belone belone*, DMM IE/16512 SL = 25.27 mm; **g** *Aplocheilus lineatus*, DMM IE/16584, SL = 3.72 mm; **h** *A. lineatus*, DMM IE/16584, SL = 6.81 mm; **i** *Aphyosemion striatum*, DMM IE/16581, SL = 2.93 mm; **j** *Epiplatys annulatus*, DMM IE/16588 SL = 3.19 mm. Abbreviations: CC, compound centrum; EO, extra caudal ossicle; EU, epural; HA, haemal arch; HS, haemal spine; HYP, hypural; IHC, inter-haemal spine cartilage; LHP, lower hypural plate; NA, neural arch; NO, notochord; NS, neural spine; PH, parhypural; PU, preural centrum; UC, ural centrum; UHP, upper hypural plate; UN, uroneural. White arrows indicate duplicated NA and NS. Scale bar: 200 μm

Along with *Glossolepis incisus* other atheriniform species were examined: *Atherina presbyter*, *Bedotia geayi* (Fig. 4a, b), *Iriatherina werneri*, *Leuresthes tenuis, Marosatherina ladigesi*, *Melanotaenia lacustris*, *Pseudomugil furcatus* (Fig. 4c, d). The development of the caudal-fin skeleton in these taxa is very similar to that found in *G. incisus*. The closely related melanotaeniid *M. lacustris* shows no differences in the development while in the other melanotaeniid *I. werneri* HYP3 distally fuses to HYP4 very late in ontogeny. During the ontogeny of the telmatherinid species *M. ladigesi* the PH does not fuse to the LHP and remains separated from the CC in adult specimens. HYP3, HYP4 and HYP5 stay separate and also do not fuse to the CC. The development of the caudal-fin skeleton of the pseudomugilid *P. furcatus* (Fig. 4c, d) differs remarkably from that of *G. incisus*. HYP1 and HYP2 do not develop as separate entities but form the LHP from earliest appearance; the upper hypural plate (UHP) in the examined developmental stages forms as a single cartilaginous element without visible separate precursors of HYP3 and HYP4; the PH develops as a portion of the LHP and is only distinguishable from it by a small proximal notch (Fig. 4c, d). During the ossification of the LHP and the UHP, both fuse onto the respective centrum developing anterior to each of them (Fig. 4d). No HYP5 is developed during ontogeny. The bedotiid *B. geayi* retains separated HYP3, HYP4 and HYP5 during ontogeny and the fusion of HYP1 and HYP2 to form the LHP happens very late in ontogeny during the ossification of these structures (Fig. 4a, b). In the atherinid *A. presbyter* the PH develops separated from the LHP and does not fuse to it. Also, HYP3, HYP4 and HYP5 remain separated and do not fuse to the CC. Similar, during the development of the atherinopsid *L. tenuis* the PH remains separated from the LHP and HYP3, HYP4 and HYP5 do not fuse.

### Beloniformes

#### Adrianichthyidae: *Oryzias woworae* (Fig. 1b, 5)

In adult specimens of *Oryzias woworae* (Fig. 1b) the caudal-fin skeleton comprises PU2, PU3, PU4 and the CC as well as the respectively associated elements. Fused to each PU are a neural arch with an elongated NS and a haemal arch with an elongated HS. The haemal spine of PU2 is more robust than the proceeding ones. The CC is characterized by its shape: the anterior portion is shaped like a half hourglass centrum, while the posterior portion is similar to an upwards-pointing cone. Ventrally the PH articulates with the CC. Postero-ventrally the LHP is fused to the CC and posteriorly the UHP is fused to the CC. A reduced neural arch is present on the CC dorsally. Antero-dorsal to the UHP two EU are present. Between the HS of PU2 and the HS of PU3 an extra caudal ossicle is present (EO). Between the distal tip of the EO and the distal tip of the HS of PU2 the IHC3 is present (not stained in Fig. 1b).

**Fig. 5:**
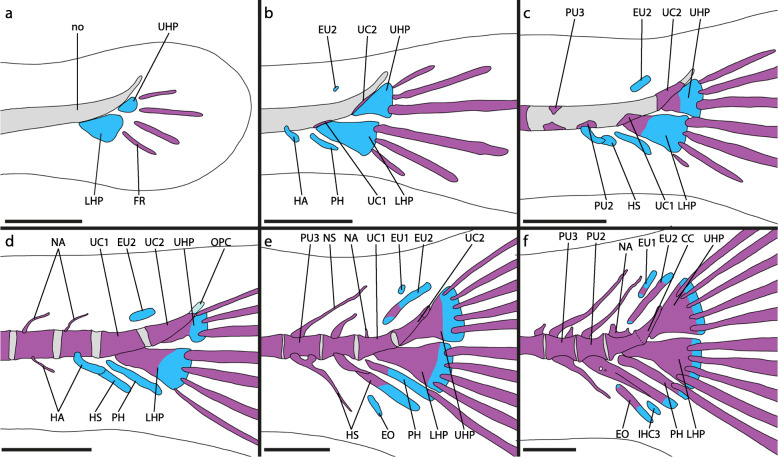
Development of caudal-fin skeleton of the beloniform *Oryzias woworae* (DMM IE/16587). **a** NL = 2.77 mm; **b** SL = 3.48 mm; **c** SL = 3.84 mm; **d** SL = 3.91 mm; **e** SL = 5.79 mm; **f** SL = 6.54 mm. Abbreviations: CC, compound centrum; EO, extra caudal ossicle; EU, epural; FR, fin ray; HA, haemal arch; HS, haemal spine; IHC, inter-haemal spine cartilage; LHP, lower hypural plate; NA, neural arch; NO, notochord; OPC, opisthural cartilage; PH, parhypural; PU, preural centrum; UC, ural centrum; UHP, upper hypural plate. Scale bar: 200 μm

The first elements of the caudal-fin skeleton to develop in *Oryzias woworae* are the cartilaginous precursors to the LHP and UHP, which appear after flexion of the notochord has started (Fig. 5a). No separate HYP1 or HYP2 and no separate HYP3, HYP4 or HYP5 are visible during development at any time. The next structures to emerge are the cartilaginous PH, which is separated from the notochord and the LHP, the cartilaginous haemal arch of PU2, and the cartilaginous EU2 (Fig. 5b). Two ossification centres representing UC1 and UC2, respectively, appear ventral to the notochord and anterior to the LHP and the UHP (Fig. 5b). The centra of PU2 and PU3 are formed in sequence with the rest of the vertebral centra and emerge slightly after the ural centra, which ossify around the notochord from ventral to dorsal (Fig. 3c, 5c). The ossification of the hypural plates starts after the formation of the ural centra and the plates immediately fuse to the respective ural centrum (Fig. 5c, d). The haemal spine of PU2 develops as an autogenous cartilage between the tips of the respective haemal arch halves. The neural arch of PU2 develops shortly after the formation of the centrum is completed (Fig. 5e). Postero-dorsal to UC2 cartilaginous cells develop at the tip of the notochord, representing the opisthural cartilage (Fig. 5d: OPC). These cells are distinct from the rest of the notochord and in later developmental stages are ossified and fused to UC2 (Fig. 5e). The parhypural ossifies and the haemal spine of PU2, which is proximally surrounded by the haemal arch of PU2, also begins to ossify and fuses to the haemal arch (Fig. 5e). EU2 has grown in relation to the previous stage and starts ossifying. Anterior to it, EU1 emerges as a small cartilage and ventrally a cartilage develops anterior to the distal tip of the haemal spine of PU2, representing the precursor of the extra caudal ossicle (Fig. 5e: EO). On the dorsal side of UC1 a neural arch develops. Both ural centra grow and thereby fill the gap between each other until they fuse to form a CC (Fig. 3d, 5f). The margins of the two UC remain visible as a fusion line. Both, EU1 and the EO, have grown and start to ossify (Fig. 5f). Between the EO and the haemal spine of PU2 the IHC3 develops. In the further course of ontogeny, the CC shrinks in proportion to the other elements and mostly the posterior portion is reduced in length. The PH grows towards the ventro-lateral margin of the CC and articulates with it. A tiny parhypurapophysis develops on the proximal part of the PH.

### Other Beloniformes

**Fig. 6:**
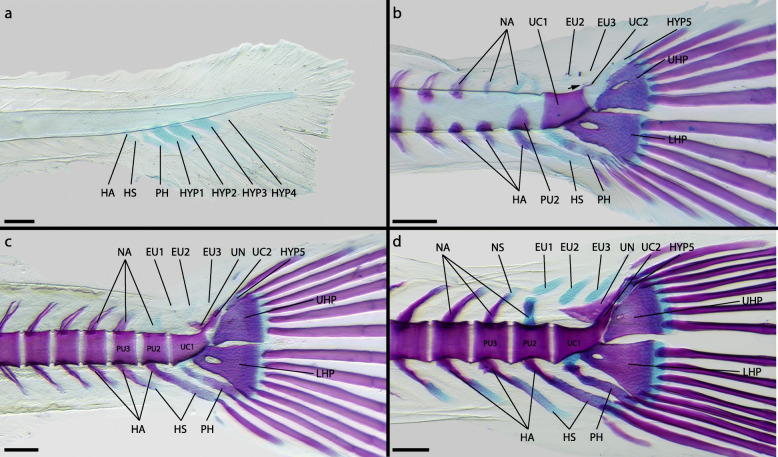
Development of the caudal-fin skeleton of the beloniform *Hyporhamphus* cf. *limbatus* (PMJ PISC-1857). **a** SL = 3.65 mm; **b** SL = 7.23 mm; **c** SL = 10.15 mm; **d** SL = 11.91 mm. Abbreviations: EU, epural; HA, haemal arch; HS, haemal spine; LHP, lower hypural plate; NA, neural arch; NO, notochord; PH, parhypural; PU, preural centrum; UC, ural centrum; UHP, upper hypural plate. Arrow points at developing uroneural. Scale bar: 200 μm

In addition to *Oryzias woworae*, late developmental stages of *Oryzias sinensis* (Fig. 4e) and *Belone belone* (Fig. 4f) as well as an ontogenetic series of *Hyporhamphus* cf. *limbatus* (Fig. 6) were available for examination. The specimens of *O. sinensis* suggest a development of the caudal-fin structures similar to that of *O. woworae* (Fig. 4e). A difference between the two adrianichthyids is the development of a reduced uroneural which is fused to the compound centrum. The ontogenetic series of *H. limbatus* (Fig. 6) indicates some differences compared to the development of *O. woworae*. In preflexion larvae, individual hypurals (i.e., HYP1, HYP2, HYP3 and HYP4) develop, which in flexion and postflexion stages fuse to form the LHP and UHP (Fig. 6a, b). HYP5 develops after the ossification of the other hypurals has already begun. After UC1 has emerged, UC2 develops from a dorsal ossification center only, which is in close contact to UC1 (Fig. 6b). Afterwards UC2 grows dorsally towards the tip of the notochord and posteriorly towards the UHP (Fig. 6c), while fusing with UC1 anteriorly. When UC2 has fully surrounded the notochord, it fuses with UC1 posteriorly (Fig. 6d). There are no traces of the margins of the two UC remaining after the fusion is completed. Additionally, a UN develops, enlarges and obtains a triangular shape (Fig. 6c, d). Three EU develop in *H. limbatus* of which the most anterior one develops after the other two (Fig. 6b, c). During the formation of the PH a cartilaginous connection between the proximal tip of the PH and the LHP is present. In *B. belone* five independent hypurals emerge before HYP1 and HYP2 fuse to form the LHP, while HYP3, HYP4 and HYP5 remain separate (Fig. 4f). The CC has already formed in the examined larval stages. Based on the shape of the CC in these larvae compared to *H. limbatus* it can be assumed that U1 and U2 developed independently and fused. An EO does not develop in *H. limbatus* and *B. belone*.

### Cyprinodontiformes

#### Procatopodidae: *Poropanchax normani* (Fig. 1c, 7)

The caudal-fin skeleton of adult *Poropanchax normani* (Fig. 1c) comprises three preural vertebrae (PU2, PU3 and PU4) and the CC. Fused to each PU are a neural arch and a haemal arch, each of which have elongated unpaired spines. The shape of the CC is characterized by an anterior portion shaped like a half hourglass and a posterior portion best described as an upward-pointing cone. Ventrally the PH articulates with the CC and posteriorly one large hypural plate (HP), with a characteristic foramen in its anterior middle portion, is fused to the CC. A small uroneural is fused to the CC dorsally. Above the CC one EU is present. In adult specimens there are 5 lower and 5 upper principal caudal-fin rays and 6–7 ventral and 6 dorsal procurrent fin rays.

**Fig. 7:**
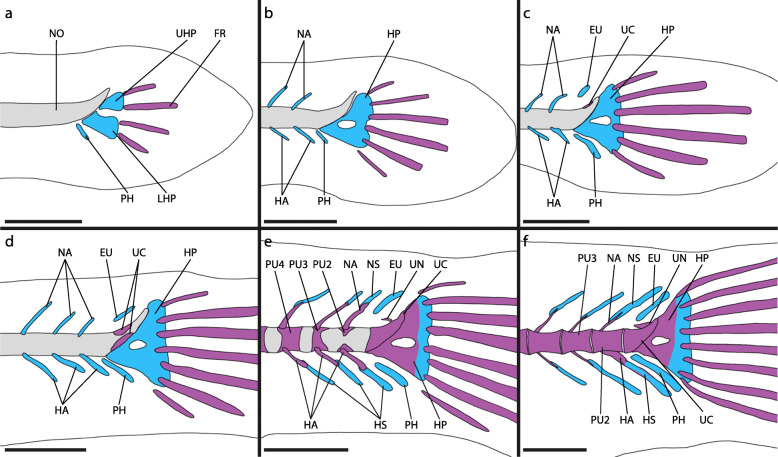
Development of caudal-fin skeleton of the cyprinodontiform *Poropanchax normani* (DMM IE/16586). **a** NL = 2.71 mm; **b** SL = 3.20 mm; **c** SL = 3.22 mm; **d** SL = 3.41 mm; **e** SL = 3.43 mm; **f** SL = 4.31 mm. Abbreviations: EU, epural; FR, fin ray; HA, haemal arch; HP, hypural plate; HS, haemal spine; LHP, lower hypural plate; NA, neural arch; PH, parhypural; PU, preural centrum; UC, ural centrum; UHP, upper hypural plate; UN, uroneural. Scale bar: 200 μm

The development of skeletal structures in the caudal fin of *Poropanchax normani* begins after flexion of the notochord has begun. First elements to emerge are the cartilaginous hypurals that represent the LHP and the UHP. Anterior to the LHP a separate cartilage, the PH, develops (Fig. 7a). As these structures then grow and first fin rays develop there are initially two fin rays associated with each hypural plate. The hypural plates grow towards each other proximally and distally and fuse, leaving a central foramen (Fig. 7b). The neural and haemal arches form as cartilaginous precursors in series from anterior to posterior. An epural emerges dorsally opposite the parhypural (Fig. 7c). Between the distal tips of the most posterior neural and haemal arches additional cartilaginous elements appear, representing autogenous neural and haemal spines. On the dorsal side of the notochord, anterior to the hypural plate, an ossification centre emerges, signalling the development of the ural centrum (Fig. 3e, 7c). While the caudal tip of the notochord shortens, the hypural plate grows dorsally filling the resulting space. Opposite the dorsal UC ossification centre, another ossification centre develops ventrally (Fig. 7d). These grow towards each other to form the ural centrum (Fig. 3f). The vertebra centra ossify from anterior to posterior, the centra of PU2 to PU4 being the last to develop. The hypural plate ossifies rapidly from anterior to posterior (Fig. 7d, e). The cartilages between the neural and haemal arches of the posterior centra grow distally and form elongated neural and haemal spines (Fig. 7e). The neural and haemal arches ossify and fuse to the respective centra (Fig. 7e, f). Dorsally on the ural centrum the paired uroneural develops and later fuses to the hypural plate. The neural and haemal spines, parhypural and epural ossify last. The autogenous neural and haemal spines fuse to their respective arch while ossifying (Fig. 7f). The PH grows dorsally towards the ural centrum and articulates with it (Fig. 1c). A parhypurapophysis develops proximo-laterally on the PH. The foramen in the hypural plate remains but gets smaller during growth. The ural centrum shortens posteriorly resulting in a half-centrum anteriorly and a dorsally pointing cone posteriorly (Fig. 1c).

### Other Cyprinodontiformes

**Fig. 8:**
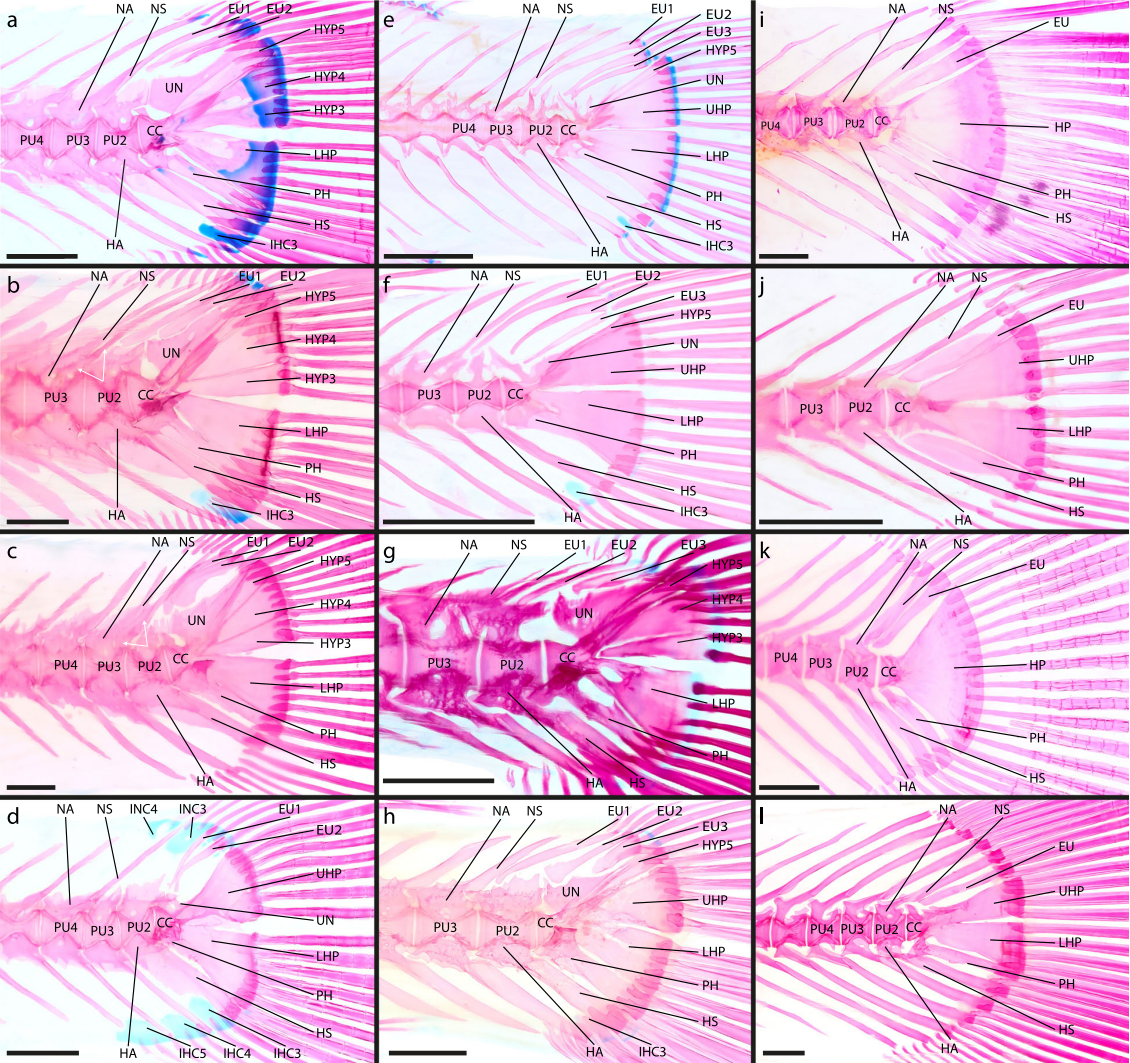
Adult caudal-fin skeleton of atheriniforms (**a-d**), beloniforms (**e-h**) and cyprinodontiforms (**i-l**). **a** *Membras martinica*, DMM IE/11398 SL = 49.0 mm; **b** *Atherina hepsetus*, DMM IE/16510 SL = 70.32 mm; **c** *Bedotia geayi*, DMM IE/16309 SL = 63.13 mm; **d** *Pseudomugil furcatus*, DMM IE/16311 SL = 41.14 mm; **e** *Dermogenys siamensis*, DMM IE/16502 SL = 27.15 mm; **f** *Nomorhamphus kolonodalensis*, ZFMK 49237–53, SL = 29.08 mm; **g** *Belone belone*, DMM IE/16519 SL = 84.72 mm; **h** *Xenentodon cancila*, DMM IE/16509 SL = 95.66 mm; **i**) *Aplocheilus lineatus*, DMM IE/16599 SL = 42.08 mm; **j** *Ameca splendens*, DMM IE/16535 SL = 37.91 mm; **k**) *Anableps macrolepis*, DMM IE/14934 SL = 68.4 mm; **l** *Aphyosemion bitaeniatum*, DMM IE/16522 SL = 20.93 mm. Abbreviations: CC, compound centrum; EU, epural; HA, haemal arch; HP, hypural plate; HS, haemal spine; HYP, hypural; IHC, inter-haemal spine cartilage; INC, inter-neural spine cartilage; LHP, lower hypural plate; NA, neural arch; NS, neural spine; PH, parhypural; PU, preural centrum; UHP, upper hypural plate; UN, uroneural. White arrows indicate duplicated NA and NS. Scale bar = 1 mm

Along with *Poropanchax normani* other cyprinodontiform species were examined: *Aplocheilus lineatus* (Fig. 4g, h), *Aphyosemion striatum* (Fig. 4i), *Epiplatys annulatus* (Fig. 4j), and *Pachypanchax omalonotus*. The caudal-fin development in cyprinodontiforms is very similar. In the aplocheilid *A. lineatus* the LHP develops as one entity and HYP3 and HYP4 develop separately before fusing later in development (Fig. 4g, h, 8i), but no HYP5 appears separately. In late developmental stages of the second examined aplocheilid *P. omalonotus* the LHP and the UHP (unclear if HYP3 and HYP4 develop separately) fuse to form one large HP. The development of the caudal-fin skeleton in the nothobranchids *Aphyosemion striatum* (Fig. 4i) and *Epiplatys annulatus* (Fig. 4j) is similar. The LHP and UHP develop as single entities respectively and then fuse anteriorly (Fig. 4i). The single ural centrum that develops first appears centered anterior to the LHP and UHP.

## Discussion

### Atheriniform caudal-fin development

The development of the caudal-fin skeleton is largely consistent throughout the examined atheriniforms. In most of the examined species, five hypurals develop as separate entities. The lower hypural plate is then formed by fusion of hypural 1 and 2. The upper hypurals (hypural 3, 4 and 5) show different grades of fusion in different species, e.g. hypural 4 and 5 fuse in *G. incisus* and hypural 3 and 4 fuse in *I. werneri* and *Atherina harringtonensis* [39]. An exception is *Pseudomugil furcatus* in which two hypural plates (lower and upper hypural plate) are present but no separate hypurals develop as individual entities during any stage of ontogeny.

In all examined atheriniforms (i.e., *Atherina presbyter*, *Bedotia geayi*, *Iriatherina werneri*, *Leuresthes tenuis, Marosatherina ladigesi*, *Melanotaenia lacustris*, *Pseudomugil furcatus*), we observed two separate ural centra in late flexion to early postflexion stages. These initially separated centra fuse in later stages to form the compound centrum. This was also reported by Parenti LR [16], who described that in developmental stages of *Phenacostethus* and *Dentatherina* two ural centra are present. Two studies, on the development of the caudal skeleton in atheriniforms, i.e., *Atherina harringtonensis* [39] and *Leuresthes tenuis* [40], did not report this detail specifically. Neither the depicted specimens of *Atherina harringtonensis* nor the description [39] gave information on the presence of two separate ossifications. This may be the result of the relatively short frame during development in which the separate ossification centers are observable and the limited material available. However, the stipplings in the drawing of the latter study ([40]: Fig. 1) indicate the formation of two separate ural centra, thereby supporting our findings. We therefore conclude that the presence of two separate ural centra during ontogeny is a general atheriniform character. Parenti LR [16] assumed that preural centrum 1 and ural centrum 1 fuse into the anterior of these centra. In our specimens, there was no sign of preural centrum 1 and we conclude that preural centrum 1 is never developed.

The PH develops as an autogenous cartilage that initially has no connection to the notochord/ural centrum 1/compound centrum or hypural 1/lower hypural plate. During development a common cartilaginous base is formed that connects the parhypural and the lower hypural plate proximally and further articulates both structures with the notochord and subsequently with ural centrum 1 and then with the compound centrum. This cartilage is later reduced and the parhypural is separated from the lower hypural plate again and articulates with the compound centrum. In few species, e.g., *G. incisus*, the parhypural fuses to the lower hypural plate distally. After the reduction of the cartilage connecting the parhypural and the lower hypural plate, the latter fuses to the compound centrum (or ural centrum 1) in all herein examined species and in *A. harringtonensis* [39].

### Beloniform caudal-fin development

The herein documented development of the caudal-fin skeleton of *Oryzias woworae* is consistent with that of *Oryzias latipes* as described by Fujita [41]. Despite the availability of several smaller specimens, we could not find separate hypural 1 and 2 and suspect that the lower hypural plate of *Oryzias* is a product of evolutionary fusion of hypural 1 and 2. An evolutionary fusion of hypural 1 and 2 therefore seems to characterize Adrianichthyidae. In the hemiramphid *Hyporhamphus* cf. *limbatus* hypural 1 and 2 develop as separate entities before they fuse to form the lower hypural plate, and we suspect a similar development occurs in *Belone belone* (Belonidae), and *Hyporhamphus sajori* (Hemiramphidae), where hypural plate 1 and 2 are already fused distally in the examined specimens [41, 42]. For *Cypselurus doederleini* (Exocoetidae) [43] and *Cololabis saira* [44] it was reported that a lower hypural plate formed by the fusion of hypural 1 and 2, but at hatching the lower hypural plate was already formed and it is unclear if hypural 1 and 2 develop separately. The character state in the grundplan of beloniforms is therefore debatable. In the evolutionary framework of Atherinomorpha either two evolutionary fusions of hypural 1 and 2 must have occurred (stem groups of Cyprinodontiformes and Adrianichthyidae) or one evolutionary fusion in the stem group of the Cyprinodontea and a subsequent separation in Belonoidei. We believe that the evolution of such a fusion is more likely than an evolutionary separation with a developmental fusion. We therefore consider the developmental pattern of separately developing hypural 1 and 2 and a subsequent fusion during development, as shown for *Hyporhamphus* cf. *limbatus,* as part of the grundplan of Beloniformes.

The components of the upper hypural plate of *Oryzias* are not that easy to determine as it could either comprise hypural 3, 4 and 5 or only hypural 3 and 4, which would include the presumption that hypural 5 is reduced [41]. In the belonids *B. belone* and *Cololabis saira* and the hemiramphids *H. sajori* and *H. limbatus* hypural 3, 4 and 5 develop separately and hypural 3 and 4 fuse to form the upper hypural plate [42, 44]. In the exocoetid *Cy. doederleini* the upper hypural plate is present at hatching and its components remain unclear [43], while in another exocoetid, *Parexocoetus mento*, two upper hypurals, presumably hypural 3 and 4, are present and fuse to form the upper hypural plate [45]. Hypural 5 is not developed in either of these two taxa. It seems likely that the upper hypural plate in *Oryzias* is a product of fusion of hypural 3 and 4 and that hypural 5 is completely reduced.

The CC in all examined *Oryzias* species is a product of the fusion of ural centrum 1 and 2. While Fujita [41] assumed that preural centrum 1 is part of the anterior ural centrum, we inferred it to comprise only ural centrum 1, as there are no signs of the occurrence of a separate preural centrum 1 during ontogeny. In *C. saira*, *Cy. doederleini* and *H. sajori* only one ural centrum supposedly develops [42, 44]. However, studying the development of *H. limbatus* we found two ural centra, which fuse to form the compound centrum. This contradicts these previous results and at least supports the assumption that in hemiramphids two ural centra are present during development. Comparing the late developmental stages of *B. belone* to *H. limbatus*, it seems possible that the compound centrum is also the product of fusion of ural centrum 1 and 2. However, the developmental data for *C. saira* contradicts this assumption, leaving the presence of two ural centra at the evolutionary base of the belonids in question. The condition in the grundplan of the Beloniformes, however, still seems to be the presence of two ural centra, as the reduction of one centrum or the evolutionary fusion of both centra seems more likely than the resurgence of one centrum within two families of beloniforms.

The development of other caudal-fin skeleton structures is similar to that of *Oryzias* and the other studied beloniform species [41–45]. Exceptions are the development of a uroneural as well as the presence of a third epural. While the latter is lacking in adrianichthyids, it is present in all other beloniforms [41–46]. A uroneural develops in all beloniforms dorsal to the posterior portion of the compound centrum. In adrianichthyids it is reduced and in *O. woworae* it is absent (Fig. 4e) [41–46]. The development of an extra caudal ossicle is restricted to Adrianichthyidae and is an autapomorphy of this family [41, 46].

### Cyprinodontiform caudal-fin development

A variation in the pattern of hypural formation was observed among the cyprinodontiform species studied herein. While in *Aplocheilus lineatus* the lower hypural plate and hypural 3 and 4 develop, only two separate elements, the lower and upper hypural plate, develop in *Aphyosemion striatum*, *Epiplatys annulatus* and *Poropanchax normani*. For *Fundulus xenicus* it is reported that only a single hypural plate develops [47]. In the examined species HYP5 is not present during any point of ontogeny. It can be assumed that in the grundplan of cyprinodontiforms hypural 5 was already reduced and that hypural 3 and 4 developed as separated entities, much like in *Aplocheilus lineatus*. A common feature of cyprinodontiform development is the development of only one ural centrum, which emerges centered anterior to the lower hypural plate and upper hypural plate/hypural 3 & 4.

### Grundplan of the caudal-fin skeleton in Atherinomorpha

**Fig. 9:**
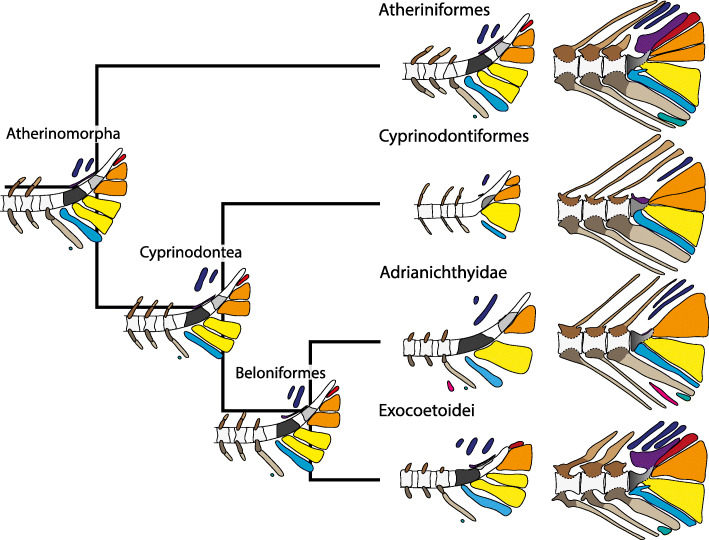
Evolution of the caudal-fin development within Atherinomorpha. A generalized scheme of hypothetical developmental stages (left column) and the adult state (right column) are shown for four studied higher atherinomorph taxa. In addition to the extant taxa, the reconstructed grundplan is drawn for each node respectively. Colour code: compound centrum, grey gradient; extra caudal ossicle, pink; epural, dark blue; haemal arch, grey-brown; haemal spine, light grey-brown; hypural 1 & 2/lower hypural plate, yellow; hypural 3 & 4/upper hypural plate, orange; hypural 5, red; inter-haemal spine cartilage 3, mint; neural arch, brown; notochord, white; neural spine, light brown; parhypural, light blue; preural centrum, light grey; ural centrum 1, dark grey; ural centrum 2, semi-light grey; ural centrum, grey; uroneural, violet

The independent development of the lower hypurals (hypural 1 and 2) is a shared character of atheriniform species [39, 40] and beloniform species [41–44]. In these taxa hypural 1 and 2 fuse to form the lower hypural plate during ontogeny (Fig. 9). In the examined adrianichthyids [41] and cyprinodontiforms the lower hypural plate seemingly develops without prior separated hypurals. As we concluded that in beloniforms and atheriniforms hypural 1 and 2 develop separately, the evolutionary fusion of hypural 1 and 2 apparently evolved in parallel in adrianichthyids and at the base of the cyprinodontiforms (Fig. 9). In the grundplan of the Atherinomorpha hypural 1 and 2 develop separately and fuse later in ontogeny. A difference in the way the lower hypural plate is developed is not evident between adult atheriniforms (Fig. 8a-d) [1, 11, 12, 14–16, 18, 20, 21, 48–52], most adult beloniforms (Fig. 8e,f,h) [1, 11, 14, 16, 53] and those adult cyprinodontiforms in which the lower and upper hypural plate are not fused (Fig. 8i-l) [11, 14, 54]. In adult specimens of the beloniform *B. belone* a foramen in the LHP indicates the fusion of two formerly separated bones (Fig. 8g).

The upper hypurals (hypural 3, 4 and 5) develop separately at the base of atheriniforms and at the base of beloniforms. At the base of the cyprinodontiforms presumably only two upper hypurals (hypural 3 and 4) develop. We conclude that in the grundplan of the Atherinomorpha three separate upper hypurals develop and that the reduction of hypural 5 occurred at the base of the Cyprinodontiformes (Fig. 9). In adult specimens separate upper hypurals persist in many atheriniform taxa (Fig. 8a-c) [20, 49]. In a few adult beloniforms, i.e., *B. belone* and *Tylosurus crocodilus*, separate upper hypurals remain [1]. In zenarchopterids and exocoetids hypural 3 and 4 are fused to form the upper hypural plate (in many species only partially) and in some species hypural 5 is part of the upper hypural plate [53]. In the scomberesocid *Cololabis saira* hypural 5 remains separated from the upper hypural plate [1, 44]. In adrianichthyids hypural 5 seems to be reduced [1, 11]. In most cyprinodontiforms an upper hypural plate is present and composed of hypural 3 and 4. Exceptions are *Aplocheilus lineatus* (some specimen) and *Epiplatys steindachneri* in which hypural 3 and 4 remain separate [11]. No hypural 5 is distinguishable in any cyprinodontiform species.

A common ontogenetic character of atheriniforms and beloniforms is the development of two ural centra that fuse to form the compound centrum during ontogeny. In cyprinodontiforms only one ural centrum develops. In the grundplan of the Atherinomorpha two ural centra develop and fuse to form the compound centrum (Fig. 9). We cannot be sure if the one ural centrum that is developed in cyprinodontiform species is the result of evolutionary fusion of both or due to the reduction of either ural centrum 1 or ural centrum 2. The position of the developing ural centrum, centered anterior to the lower and upper hypural plate, would support the first case, as in atheriniforms and beloniforms ural centrum 1 and ural centrum 2 develop anterior to the lower hypurals and upper hypurals respectively. The fusion of the two ural centra could be expected to develop in an intermediate state. If the second case applies, it would be impossible to unequivocally homologize the developing ural centrum with either ural centrum 1 or ural centrum 2 in atheriniforms and beloniforms. In adult specimens of all three taxa, the compound centrum of atheriniforms and beloniforms and the ural centrum of cyprinodontiforms are not distinguishable by their shape, which can be described as an anterior half centrum and a posterior upward-pointing cone (Fig. 8). This would also support the hypothesis that the ural centrum of cyprinodontiforms is the result of evolutionary fusion. A preural centrum 1 is neither developed separately in any of our examined species nor in any of the previously studied species [39–44, 47]. Although it was hypothesised by some authors that preural centrum 1 is part of the compound centrum in some species, we found no evidence that would support this hypothesis.

Further similarities of atheriniforms, beloniforms and cyprinodontiforms which are also part of the grundplan of the Atherinomorpha include the autogenous development of the parhypural and the epurals as well as the autogenous development of at least the haemal and neural spines of preural centra 2–5 (Fig. 9).

To recap, the grundplan of the caudal-fin development of the Atherinomorpha includes: 1) development of five individual hypurals of which hypural 1 and 2 subsequently fuse to form the lower hypural plate; 2) development of two separate ural centra which fuse to form the compound centrum; 3) absence of preural centrum 1 during ontogeny, 4) development of an autogenous parhypural and autogenous haemal spines and neural spines of at least preural centra 2 to 5; 5) development of two autogenous epurals and 6) development of inter-haemal spine cartilage 3 (Fig. 9).

### Comparison to ovalentarian taxa

The Atherinomorpha have been considered a monophyletic group throughout the last 60 years [10, 12, 13, 16, 17, 19, 20, 22–25, 27–29, 55] but their phylogenetic position within Percomorphacea and their closest relatives remain uncertain, due to morphological [e.g., 13, 16, 56] and molecular [e.g., 25, 26, 28, 30, 31] analyses repeatedly retrieving varying results. Recently, Wainwright PC, Smith WL, Price SA*,* et al. [26], Betancur-R R, Broughton RE, Wiley EO*,* et al. [27] and Betancur-R R, Wiley EO, Arratia G*,* et al. [28] provided convincing molecular evidence for the Atherinomorpha as part of the Ovalentaria. The Ovalentaria-hypothesis suggests that many taxa, which previously were widely separated within the Percomorphacea, are closely related and form a monophylum and, therefore, provides new impulses for comparative analyses. Although molecular support values for the Ovalentaria are persuasive, the support values for ovalentarian intrarelationships for most cases are quite low. Possible sister-taxa relationships previously suggested for atherinomorphs by morphological and molecular data include the Mugilidae [13, 19, 20, 22, 24, 56], the Blennioidei and Gobiesocidae [23], the Cichlidae, Embiotocidae and Pomacentridae [25]. Recent studies suggest that the Cichlidae [29] or the group comprising Cichlidae, Polycentridae and Pholidichtyidae [27, 28] are more closely related to the Atherinomorpha.

For many of the contemplable taxa studies on the development of the caudal fin are scarce or missing. For blenniids [57], cichlids [58, 59] and clinids [60, 61] there is some ontogenetic data, and for mugilids [62] and pomacentrids [63] detailed descriptions are available. Similarities between the caudal-fin development of these taxa and the Atherinomorpha include autogenous development of the parhypural and the epurals, the autogenous development of some haemal and neural spines of the preural centra (i.e., preural centra 2 and 3 in mugilids and at least preural centra 2 and 3 in blenniids, cichlids and pomacentrids) [59, 60, 62, 63]. In the cichlids examined for this study (*Amatitlania nigrofasciata*, *Geophagus* sp., *Hemichromis bimaculatus*), the haemal spines and neural spines of preural centrum 2 and preural centrum 3 develop autogenously. A cartilaginous bridge connects the proximal tip of the parhypural to the proximal tip of hypural 1 during ontogeny in atheriniforms. Such a connection is also present in cichlids, clinids, mugilids and pomacentrids [57, 59, 62, 63] suggesting that at the base of the Atherinomorpha such a connection was present and was reduced within beloniforms and cyprinodontiforms.

At the base of the Atherinomorpha five hypurals are present during development and hypural 1 and 2 fuse to form the lower hypural plate. Five hypurals also can be seen during ontogeny in cichlids, some clinids, e.g., *Clinus cottoides*, mugilids and pomacentrids [59, 60, 62, 63]. While in cichlids no hypural fusion occurs, and the hypurals remain separate in adults, hypural 1 and 2 fuse to form the lower hypural plate in clinids, mugilids and pomacentrids. In clinids this fusion occurs early in development and additionally the parhypural fuses to the lower hypural plate. The fusion of the lower hypurals to form the lower hypural plate could be a character that positions the clinids, mugilids and pomacentrids closer to the Atherinomorpha. Fusion of the upper hypurals happens in clinids and mugilids, where hypural 3 and 4 fuse to form the upper hypural plate. Although such a fusion occurs in beloniforms and cyprinodontiforms too, it seems likely that this trait evolved independently within the atherinomorphs and clinids/mugilids based on the well supported monophyly of the Atherinomorpha. In blenniids, the lower and upper hypural plate develop without separate hypural-precursors [57]. Apparently, this is also a separately acquired character in blenniids and cyprinodontiforms.

The compound centrum in atherinomorphs develops by fusion of ural centrum 1 and ural centrum 2. Within the Ovalentaria a similar development is only known in mugilids, wherein ural centrum 1 emerges anterior to the lower hypurals and ural centrum 2 anterior to the upper hypurals and both fuse to form a compound centrum with an identical shape to the compound centrum of atherinomorphs [62]. In the other previously studied ovalentarian taxa, only one elongated ural centrum develops that covers the notochord from the beginning of the parhypural almost to the caudal tip of the notochord [57–61, 63]. During ontogeny this centrum also shortens and in adults has a similar shape as in atherinomorphs and mugilids [1]. The similar development in atherinomorphs and mugilids could indicate a closer relationship of these taxa or a shared plesiomorphic character absent in the remaining ovalentarians. The development of the ural centrum in the other taxa in contrast raises the question if this is the result of evolutionary fusion of two previously separated centra or if one ural centrum got reduced and the remaining centrum elongated and took the former’s place. The connection of these two developmental modes remains unanswered for now and needs more detailed developmental studies of a variety of ovalentarian taxa to be answered with more certainty. Subsequently, this would help to evaluate the validity of the Ovalentaria based on morphological data.

## Conclusion

At the base of atheriniforms and beloniforms five hypurals develop, of which hypural 1 and hypural 2 fuse to form the lower hypural plate, while only the lower hypural plate and two upper hypurals develop at the base of cyprinodontiforms. The development of the compound centrum is very similar in atheriniforms and *Oryzias*, wherein two ural centra develop and fuse to form the compound centrum, whereas in the other studied beloniforms and in cyprinodontiforms only one centrum develops. The reduction of one centrum or the evolutionary fusion of the two centra must have occurred independently within beloniforms and in cyprinodontiforms based on the phylogenetic relationships within atherinomorphs provided by both morphological and molecular data. The grundplan of a last common ancestor to all atherinomorphs is very much similar to that of extant atheriniforms. Comparing the caudal-fin development of atherinomorphs to that of other ovalentarian taxa, we found most similarities with mugilids, which develop five separate hypurals of which hypural 1 and hypural 2 fuse, two ural centra, which fuse, and an autogenous parhypural that is connected to hypural 1 by a cartilaginous bridge.

## Data Availability

All specimens examined in this study are included in the Material and Methods section of this publication. Raw images used for drawings are available upon request from the first author.
